# Scorepochs: A Computer-Aided Scoring Tool for Resting-State M/EEG Epochs

**DOI:** 10.3390/s22082853

**Published:** 2022-04-08

**Authors:** Matteo Fraschini, Simone Maurizio La Cava, Giuseppe Rodriguez, Andrea Vitale, Matteo Demuru

**Affiliations:** 1 Department of Electrical and Electronic Engineering, University of Cagliari, 09124 Cagliari, Italy; s.lacava@studenti.unica.it; 2 Department of Mathematics and Computer Science, University of Cagliari, 09124 Cagliari, Italy; rodriguez@unica.it; 3 Laboratory for Autism and Neurodevelopmental Disorders, Center for Neuroscience and Cognitive Systems @UniTn, Istituto Italiano di Tecnologia, 16163 Rovereto, Italy; andrea.vitale@iit.it; 4 Liceo Scientifico e Linguistico Statale “G. Marconi”, 07100 Sassari, Italy; matteo.demuru@posta.istruzione.it

**Keywords:** M/EEG, epoch selection, resting-state

## Abstract

M/EEG resting-state analysis often requires the definition of the epoch length and the criteria in order to select which epochs to include in the subsequent steps. However, the effects of epoch selection remain scarcely investigated and the procedure used to (visually) inspect, label, and remove bad epochs is often not documented, thereby hindering the reproducibility of the reported results. In this study, we present Scorepochs, a simple and freely available tool for the automatic scoring of resting-state M/EEG epochs that aims to provide an objective method to aid M/EEG experts during the epoch selection procedure. We tested our approach on a freely available EEG dataset containing recordings from 109 subjects using the BCI2000 64 channel system.

## 1. Introduction

The use of task-free resting-state M/EEG (magneto-electroencephalogram) recordings represents one of the most used experimental paradigms to investigate the baseline level of brain activity in healthy subjects and patients [1]. However, the resting-state condition is an elusive concept influenced by different states of vigilance that are usually out of the control of the experimenter [2]. Generally, the first steps performed during an M/EEG resting-state analysis consist of (i) segmenting the raw and filtered EEG traces into a set of non-overlapping epochs and (ii) selecting a number of artifact-free epochs to be used during the subsequent steps of the pipeline. These steps require the definition of the epoch length and the criteria to select which epochs to include in the successive analysis. The effects of the epoch length have been previously investigated [3], while the effects of epoch selection, induced by the inter-observer variability and unclear criteria used for this task, remain scarcely investigated. Epoch selection is performed at the individual level (independently for each subject) and is usually conducted by one or more experts. Some kind of procedure to detect and mitigate EEG artifacts may be applied before this step. However, the precise procedure used to (visually) inspect, label, and remove bad epochs is often not documented [4], thereby hindering the reproducibility of the reported results. Most importantly, especially if the selection procedure is performed by different experts, it would be of relevance to assure that homogeneous criteria were used. In this context, most of the studies using resting-state paradigms make assumptions on the stationarity of EEG signals and perform averaging of individual features (extracted at the subject level) to make inferences at the group level. In short, this means that strong within-subject stability of M/EEG features is assumed and that these individual characteristics may be consistent within a group. Subjective visual scoring and inter-observer variability pose possible threats to the validity of these assumptions, although some studies have reported that the subjective influence may lead to minimal changes when a sufficient number of epochs are selected [5,6]; nevertheless, it is still unclear how to quantify this sufficient number of epochs. In this context, a very important role will be assumed with the possibility of developing some kind of semi-automated analysis with the aim of helping clinicians and researchers during these very crucial steps. Few recent studies have used computer-assisted tools to allow EEG background patterns to be interpreted [7,8]; however, as suggested by van Diessen et. al. [4], these methods have not been applied at large scales because of the inherent complexity or limited transparency. On the other hand, other studies have suggested the use of automatic artifact suppression [9], which would indirectly help to limit the uncertainty induced by inter-observer variability. Most of these approaches are based on independent component analysis (ICA) [10], which requires a great amount of EEG data to achieve acceptable decomposition (a minimum of 20 time points per channel^2) [11]. In this study, we present Scorepochs, a simple and freely available tool for the automatic scoring of resting-state M/EEG epochs that aims to provide an objective method to aid M/EEG experts during epoch selection. Our approach, which works at the subject level, provides a score for each epoch within a single M/EEG recording, as an attempt to make this crucial procedure less ambiguous, more objective, and reproducible. It is well recognized that neural oscillations play an important role in characterizing behavioral and cognitive states [12,13] and that they are also implicated in most brain disorders. To date, spectral analysis represents the most important and commonly used tool for the characterization of neurophysiological signals [14,15]. In this context, Scorepochs is based on the whole power spectrum of the EEG, does not require any specific assumption of the underlying frequency content, and may keep all of the relevant spectral information contained in the unfiltered raw signal [16].

## 2. Methods

### 2.1. Scorepochs

**Figure 1:**
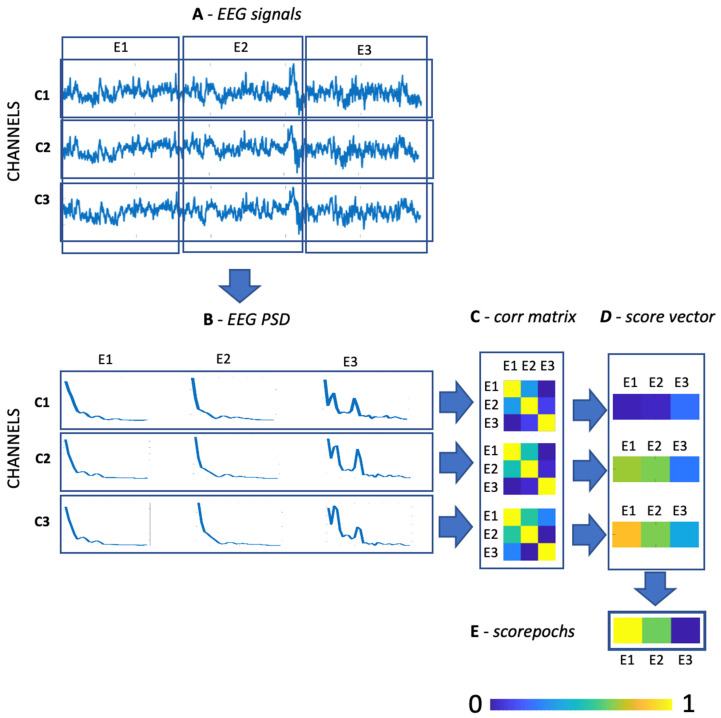
A schematic representation of the algorithm used to compute Scorepochs: (**A**) EEG raw signals of three channels with an epoch scheme; (**B**) power spectral density plots for each channel and each epoch; (**C**) correlation matrices for each channel; (**D**) score vector for each channel and for each epoch; (**E**) score for each epoch.

The proposed method is based on a very simple and fast algorithm that takes as the input (i) a set of M/EEG recordings and (ii) the length of the desired epoch. After this, the algorithm provides as the output a score for each single M/EEG epoch. A schematic representation of the proposed method is depicted in Figure 1. Furthermore, all the scripts used to perform the analysis are freely available for MATLAB (https://github.com/Scorepochs-tools/scorepochs_mat, accessed on 1 February 2022) and for Python (https://github.com/Scorepochs-tools/scorepochs_py, accessed on 1 February 2022). For each subject, each epoch, and each channel, the algorithm computes the power spectral density (PSD) via the Welch method into a specific range of frequencies (see Figure 1A,B). At the channel level, a similarity score, computed by using the Spearman correlation coefficient, is evaluated between the PSD values extracted from all the epochs, thereby providing a correlation matrix with *number of epochs x number of epochs* as a dimension (see Figure 1C). First, the average is computed over the rows (columns) of the symmetric matrix to obtain a *score vector* with a length equal to the *number of epochs*, where the entries represent the mean similarity score of the corresponding epoch (see Figure 1D). By computing the *score vector* for each channel, and then averaging the *score vectors* across channels, it is possible to obtain a final score for each epoch (see Figure 1E). Finally, for each subject, the score can be sorted in descending order allowing one to select the suggested epochs to be included in the subsequent steps of the analysis.

### 2.2. Experimental Setup and Statistical Analysis

We tested our approach on a freely available EEG dataset [17,18] containing recordings from 109 subjects collected using the BCI2000 64-channel system (http://www.bci2000.org, accessed on 1 February 2022). The EEG dataset is available at the following link: https://physionet.org/content/eegmmidb/1.0.0/, accessed on 1 February 2022. We decided to define an easily interpretable hypothetical scenario where the aim was to contrast two different baseline conditions, namely eyes open (EO) and eyes closed (EC) resting-state conditions. To contrast the two conditions, the relative alpha power (computed in the range between 8 and 13 Hz) was the perfect candidate, as this property is a very common yet effective feature able to detect macroscopic differences between EO and EC conditions. The analysis was performed on 99 out of the 109 subjects, since some of them were excluded due to differences in recording parameters or overall poor quality. We used an epoch length of 5 s and segmented the one-minute available recordings into twelve non-overlapping epochs (the results were successively replicated using two different epoch lengths of 2 and 8 s). For each epoch and each channel we extracted the relative alpha power, and the average across channels was successively evaluated for each epoch, meaning the relative alpha power was computed at the global level. To mimic a realistic epoch selection procedure and investigate its possible effect, we decided to select for each subject four of the twelve available epochs, considering 495 different selections, representing all of the possible combinations obtained by using the same subset of epochs for all subjects. In particular, 495 possible combinations can be obtained by taking a sample of items (the 4 selected epochs) from a larger set (the 12 epochs available in the dataset). We then compared the results in terms of the magnitude of the effect size obtained by contrasting the two conditions (EO vs. EC) on a group level. We computed 495 *t*-tests (assuming normality distribution, which is a limitation in this specific case: see Supplementary Material), where for each test we selected 4 epochs for every subject in sequential order (i.e., for all subjects; for the first test the epochs selected were (1, 2, 3, 4), for the second test the epochs selected were (1, 2, 3, 5), for the third test the epoch selected were (1, 2, 3, 6), …, up to the last test where the selected epochs were (9, 10, 11, 12)). We then compared the magnitude of the paired Cohen’s d effect size obtained using the selection suggested by Scorepochs against the distribution of Cohen’s d effect size based on the sequential random selection. The analysis was later replicated using a completely different method, namely the phase lag index (PLI) [19], to compare the two experimental conditions. We performed this further analysis to investigate whether the proposed approach might be successfully applied to different methods.

### 2.3. A Comparison with ICLabel Algorithm

Finally, to understand how much Scorepochs reflects the selection of good epochs (and not merely driven by recurring artifacts), we compared the proposed approach with another potential method based on the independent component classification (the number of components identified as neural is generally considered a reliable estimate of EEG signal quality [20]). For this purpose, we used the ICLabel algorithm [21], which provides—for every single component—the probability of having a cortical generator or belonging to an artifactual class (muscular, ocular, or other artifacts). Each component with a probability >20% of having a neural source was assigned a “brain” class. The number of “brain” components was correlated—at the single-subject level—with the scores averaged across all epochs (in this case, 30 epochs of 2 s length in the 1–40 Hz spectrum) as computed by Scorepochs during the eyes open condition. We conducted this comparison for two different possible preprocessing scenarios. The first scenario (pipeline_01) included the use of a bad-channel rejection approach, namely “cleanrawdata” [22], together with an artifact detection and repair method [23]. The second scenario (pipeline_02) included the use of >3 standard deviations for bad channel rejection together with wavelength-enhanced ICA [24] for the artifact detection procedure. Later, we computed a robust statistical measure of association between the two parameters (Scorepoches vs. ICLabel output) by down-weighting the outliers [25].

## 3. Results

**Figure 2:**
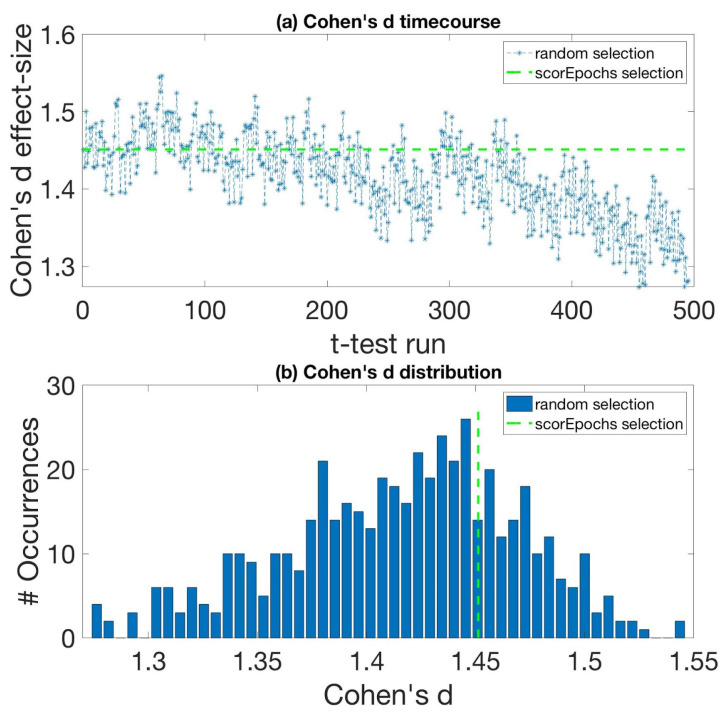
Cohen’s d effect sizes for random selection and Scorepoch selection using a time window equal to 5 s. (**a**) The ‘time course’ of the effect size computed using a sequential random selection. The effect size values are reported in the y-axis, while the x-axis indicates the *t*-test with sequential random selection. The green dashed line represents Cohen’s d value obtained by selecting the epochs using Scorepochs. (**b**) Cohen’s d effect size distribution for the random epoch selection and the Scorepochs selection. The effect size values are reported on the x-axis, while the y-axis indicates the occurrences of specific effect size values. The vertical green dashed line represents Cohen’s d value from selecting the 4 epochs suggested by Scorepochs.

All of the results from the comparison of the magnitude of the paired Cohen’s d effect size obtained using the selection suggested by Scorepochs against the distribution of Cohen’s d effect size based on the sequential random selection are summarized in Table 1 and Figure 2, which show the comparison, in terms of Cohen’s d effect size values, between the described sequential random selection and the selection suggested by our approach. In particular, Figure 2a depicts the ‘effect size time course’ using this random selection together with the result derived from the application of our method, represented by the green dashed line. A decreasing trend in terms of effect size values can be observed. Figure 2b shows the distribution of the effect size values (independently of the sequential order), whereby the vertical green dashed line represents the value of the effect size obtained using the epochs suggested by our approach. The Cohen’s d value for the Scorepochs algorithm is 1.4512, which is around the 75th percentile of the random epoch selection distribution. It is worth noting that the minimum effect size is bigger than 1, meaning that as expected, the difference is largely independent of the epoch selection strategy (i.e., the difference between the two conditions EO and EC is reliably measurable).

**Figure 3:**
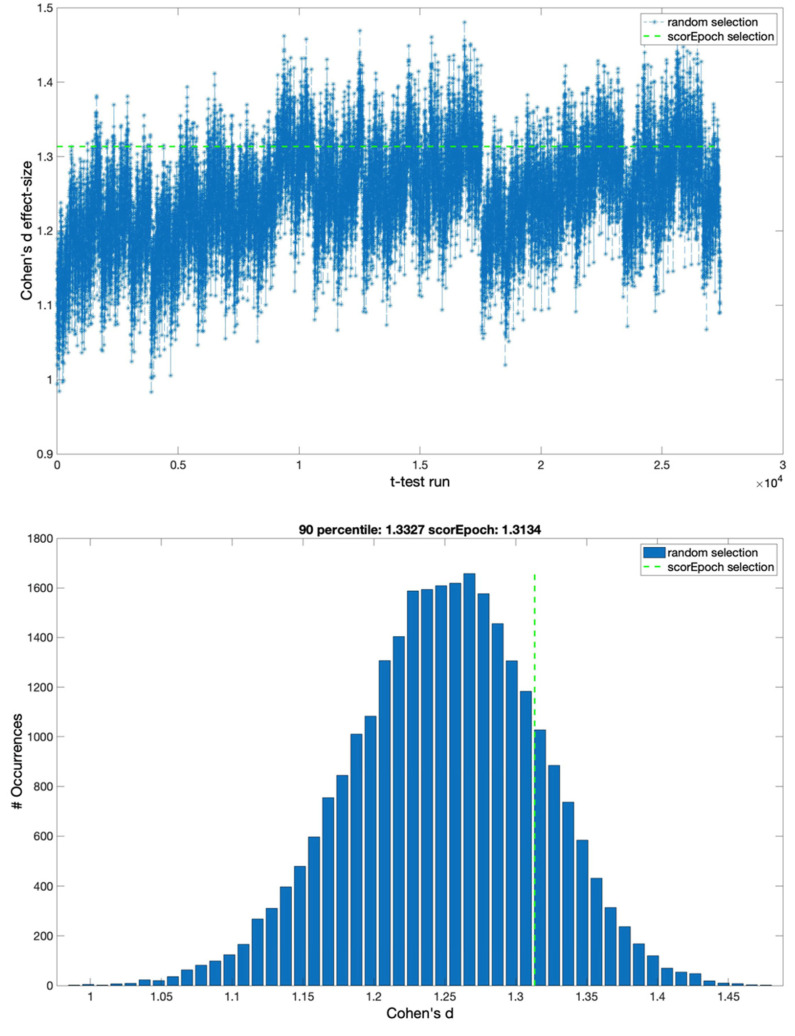
Cohen’s d effect sizes for random selection and Scorepoch selection using a time window equal to 2 s. (**upper panel**) The ‘time course’ of the effect size computed using a sequential random selection. The effect size values are reported in the y-axis, while the x-axis indicates the *t*-test with a sequential random selection. The green dashed line represents Cohen’s d value obtained by selecting the epochs using Scorepochs. (**lower panel**) Cohen’s d effect size distribution for the random epoch selection and the Scorepoch selection. The effect size values are reported on the x-axis, while the y-axis indicates the occurrences of specific effect size values. The vertical green dashed line represents Cohen’s d value from selecting the 4 epochs suggested by Scorepochs.

**Figure 4:**
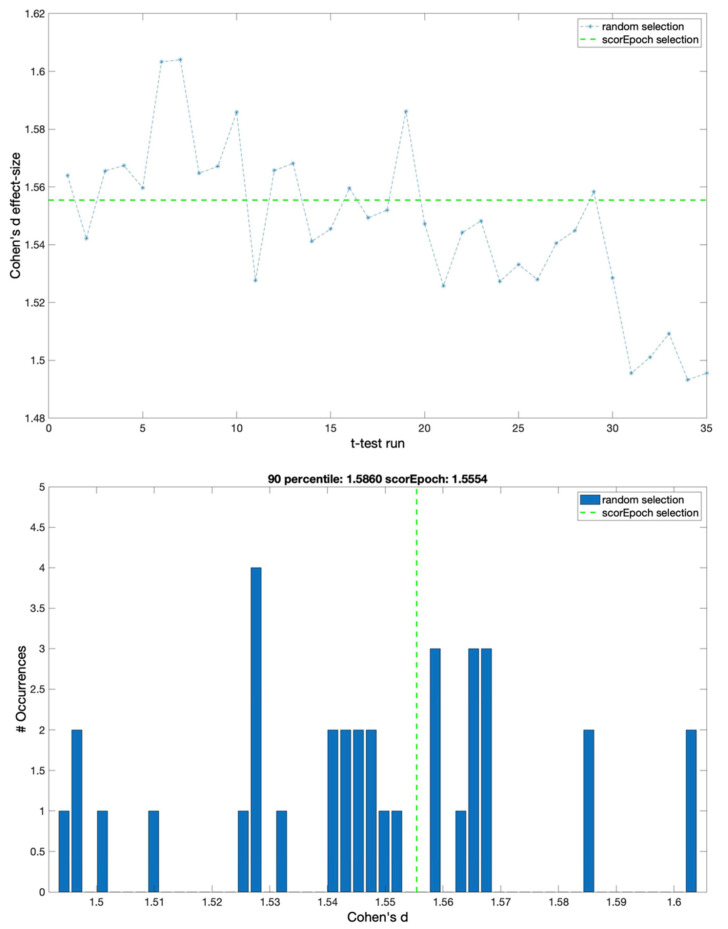
Cohen’s d effect sizes for random selection and Scorepoch selection using a time window equal to 8 s. (**upper panel**) The ‘time course’ of the effect size computed using a sequential random selection. The effect size values are reported in the y-axis, while the x-axis indicates the *t*-test with a sequential random selection. The green dashed line represents Cohen’s d value obtained by selecting the epochs using Scorepochs. (**lower panel**) Cohen’s d effect size distribution for the random epoch selection and the Scorepoch selection. The effect size values are reported on the x-axis, while the y-axis indicates the occurrences of specific effect size values. The vertical green dashed line represents Cohen’s d value selecting the 4 epochs suggested by Scorepochs.

With the aim of investigating the possible effects of the time window on the reported results, we reproduced the analysis using two different epoch lengths of 2 and 8 s. The results obtained from this new analysis are represented in Figure 3 and Figure 4.

**Figure 5:**
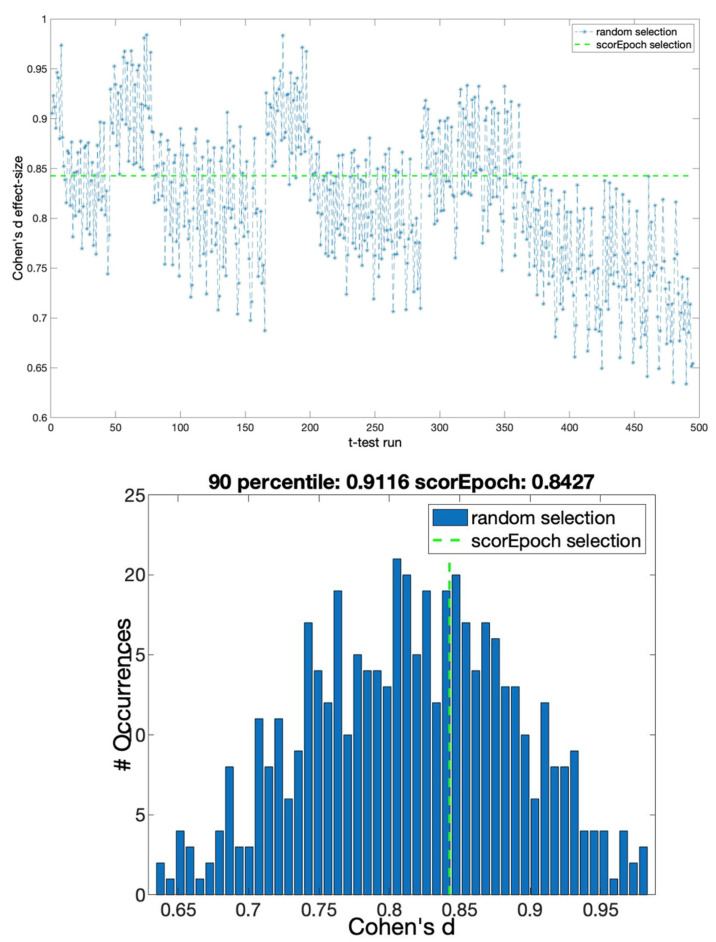
Cohen’s d effect sizes for random selection and Scorepoch selection using a time window equal to 5 s for the PLI method. (**upper panel**) The ‘time course’ of the effect size computed using a sequential random selection. The effect size values are reported in the y-axis, while the x-axis indicates the *t*-test with a sequential random selection. The green dashed line represents Cohen’s d value obtained by selecting the epochs using Scorepochs. (**lower panel**) Cohen’s d effect size distribution for the random epoch selection and the Scorepoch selection. The effect size values are reported on the x-axis, while the y-axis indicates the occurrences of specific effect size values. The vertical green dashed line represents Cohen’s d value selecting the 4 epochs suggested by Scorepochs.

The results derived from the application of the PLI method are summarized in Figure 5.

**Figure 6:**
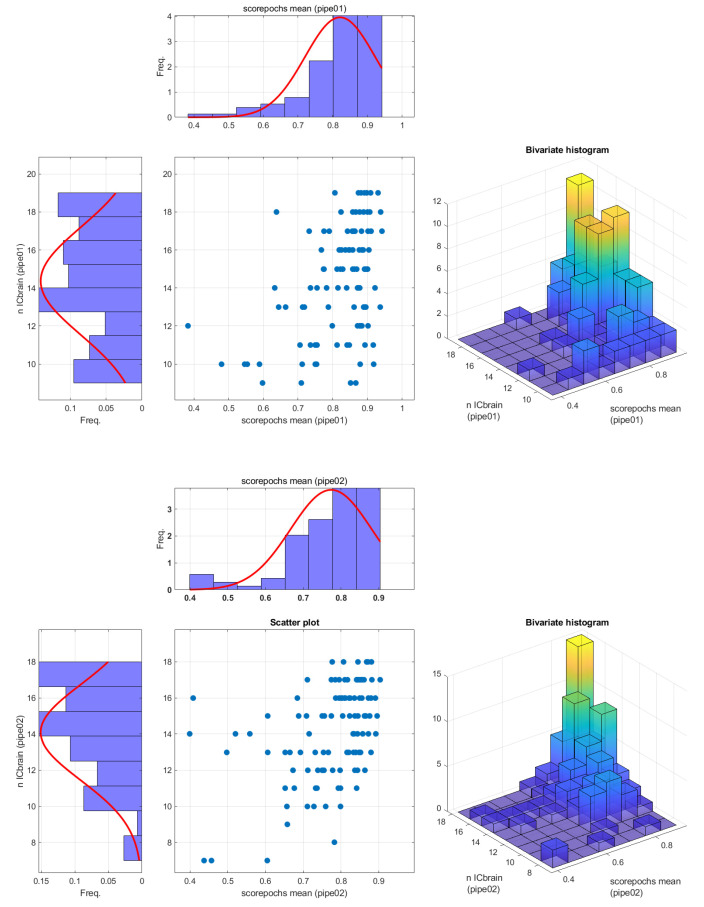
Scatterplots representing the association between the scores computed by Scorepochs and the number of “brain” components as defined by the ICLabel algorithm for pipeline_01 (**upper panel**) and pipeline_02 (**lower panel**).

The last part of the analysis, as summarized in Figure 6, provides an indirect validation of the effectiveness of Scorepochs to select epochs while preserving brain activity. The comparison at the single subject level between Scorepochs and the number of independent components classified as “brain” shows a dense distribution in the upper-right portions of the scatter plots and bivariate histograms (for both the pipelines), whereby the higher scores correspond to a larger number of brain components. When further assessed with a robust statistical procedure such as Spearman’s skipped correlation [25], Scorepochs presents a positive association with the independent components of the brain source obtained through both preprocessing pipelines (r = 0.3604, CI = [0.1780 0.5261], t = 3.9967 for the pipeline_01 and r = 0.4371, CI = [0.2856 0.5688], t = 5.0267 for pipeline_02). A similar result was reproduced when considering a stricter threshold for classifying a component as “brain” (probability > 50%).

## 4. Discussion

In this study, we proposed an automatic method to assist M/EEG experts during the epoch selection procedure in resting-state analysis. Our method represents an objective (or less subjective) approach to performing epochs selection if compared to the potential arbitrariness introduced by human observers and the lack of clear and shared criteria used to accomplish this crucial task. We have shown in a prototypical scenario of a group comparison between two resting-state conditions (EO vs. EC) that the effect size varied extensively depending on the epochs included in the analysis. In fact, even if it is possible to detect an effect between the two conditions EO and EC almost independently of the selected epochs (since the detection of an effect is highly probable as Cohen’s d > 1 for every epoch selection), there is considerable variation in the effect size depending on the actual selection. Specifically, we showed a decreasing trend (see Figure 2a) in terms of the effect size with respect to the time in which the selection occurred (i.e., selecting epochs at the beginning of the experiment gives a higher effect size compared to the end of the experiment). This trend may be influenced by different states of vigilance related to tiredness or drowsiness that are reflected in the recorded signals. The magnitude of the effect size obtained using our proposed epoch selection was close to the mean effect size (which should represent the best estimate of the population effect size), and more importantly was based on a quantifiable, objective, and replicable strategy (i.e., scores computed on the PSD). It is also relevant to highlight that our results were successfully replicated using different sizes of the time window. Finally, our results suggest that the proposed approach may be easily extended to other methods, such as the one based on connectivity metrics. Compared to other semi-automatic procedures for the selection of the ‘best artifact-free epochs’ suitable for the analysis (e.g., independent component analysis, ICA), our method is completely data-driven, and it does not require any intervention or particular skills of the user as compared to other selection strategies (e.g., knowledge of stereotypical EEG pattern related to artifact components using ICA). Moreover, the Scorepochs method, because of the small number of requirements (i.e., computation of the PSD), is not computationally expensive. Despite its simplicity, this method is well grounded in physiological terms. In fact, it has been shown how the computation of simple statistics based on the PSD reflects intrinsic properties of excitatory or inhibitory levels of neuronal populations [26]. Furthermore, the PSD is able to capture different dynamics modulated by external stimuli and provides insights into sensory neural representation [27]. Finally, it has been recently reported how different behavioral states are reflected in different properties of the PSD [28]. As for the approach to evaluate the spectral parameters, it has been shown [14] that different spectral analysis approaches (e.g., Fourier, Hilbert, and wavelet transform approaches) yield equivalent results in practical applications. We indirectly validated the effectiveness of Scorepochs to select good epochs by comparing our method with the number of independent components classified as “brain” using ICLabel [21], an automated electroencephalographic independent component classifier. The observed robust correlation with this approach confirms that Scorepochs may provide an objective procedure for evaluating the impacts of alternative preprocessing pipelines in large-scale studies [29]. In no way should the proposed approach replace the work to be performed by experts (alone or using other automatic or semi-automatic methods) during visual inspections of real M/EEG data. Scorepochs guided selection should be complementary to the human activity or to any other selection method.

## Figures and Tables

**Table 1:** The statistics for all comparisons between random and Scorepochs selections.

Random vs. Scorepochs Selection		
Time Window	Cohen’s Effect Size for Scorepochs	Cohen’s Effect Size at 90th Percentile
5 s	1.45	1.48 at 90th
2 s	1.31	1.33 at 90th
8 s	1.56	1.58 at 90th

## Data Availability

In this study a freely available EEG dataset was used. The dataset is available at the following link: https://physionet.org/content/eegmmidb/1.0.0/ (accessed on 1 February 2022).

## Supplementary Materials

The following supporting information can be downloaded at: https://www.mdpi.com/article/10.3390/s22082853/s1, Figure S1: Non-parametric test.
