# Supplementary material for: Scorepochs: A Computer-Aided Scoring Tool for Resting-State M/EEG Epochs
Source: Sensors (Basel). 2022 Apr 8;22(8):2853. doi: 10.3390/s22082853 (PMC9031998; doi:10.3390/s22082853)
Supplement: Supplementary file 1 [file sensors-22-02853-s001.zip › sensors-1624000-supplementary.pdf]

Scorepochs: A Computer-Aided Scoring Tool for Resting-State M/EEG Epochs

Matteo Fraschini [1,*], Simone Maurizio La Cava [1], Giuseppe Rodriguez [2], Andrea Vitale [3] and Matteo Demuru [4]

[1] Department of Electrical and Electronic Engineering, University of Cagliari, Cagliari, Italy
[2] Department of Mathematics and Computer Science, University of Cagliari, Cagliari, Italy
[3] Laboratory for Autism and Neurodevelopmental Disorders, Center for Neuroscience and Cognitive Systems @UniTn, Istituto Italiano di Tecnologia, Rovereto, Italy
[4] Liceo Scientifico e Linguistico Statale "G. Marconi", Sassari, Italy

Non-parametric test

In order to understand the possible effect induced by the statistical test, we have performed the analysis using the Wilcoxon signed rank test and computing the effect size as r = Z / sqrt(N), see Pallant, 2007.

As expected, the results are perfectly in line with the previous findings:

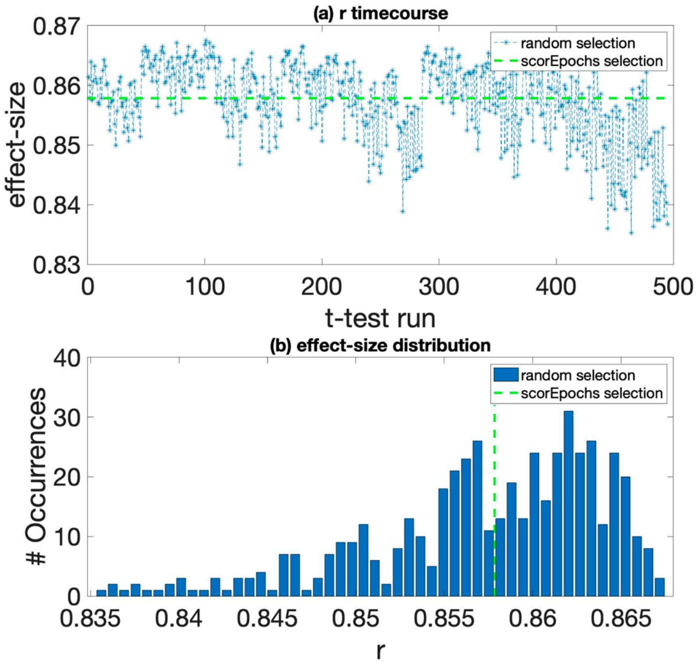

Figure S1. Non-parametric test
